# *haploR*: an R package for querying web-based annotation tools

**DOI:** 10.12688/f1000research.10742.2

**Published:** 2017-05-15

**Authors:** Ilya Y. Zhbannikov, Konstantin Arbeev, Svetlana Ukraintseva, Anatoliy I. Yashin

**Affiliations:** 1 Biodemography of Aging Research Unit (BARU) at Social Science Research Institute, Duke University, Durham, NC, USA; 2 Duke Population Research Institute, Duke University, Durham, NC, USA

**Keywords:** R, databases, genomics, genetic variants, genome annotation, data mining

## Abstract

We developed *haploR*, an R package for querying web based genome annotation tools HaploReg and RegulomeDB. *haploR* gathers information in a data frame which is suitable for downstream bioinformatic analyses. This will facilitate post-genome wide association studies streamline analysis for rapid discovery and interpretation of genetic associations.

## Introduction

Genome wide association studies (GWAS) have produced a significant amount of data. To better understand the biological mechanisms involved in complex trait regulations, web-based tools, such as HaploReg ^1^ and RegulomeDB ^2^, were proposed. These tools offer a link of detected genetic variants to additional post-GWAS information about linkage disequilibrium (LD), expression quantitative trait loci (eQTL), allele frequencies (AF), protein functions, and chromatin states (for annotated single-nucleotide polymorphisms (SNP)). These tools are all web-based and require the user to do the following: open a web page, manually enter information, and obtain the results. The user needs to advise that in a number of situations, extra precautions must be made. Two examples of this would be saving the results in different file formats (TXT, CSV, XLSX, etc.,) or taking advantage of their highly-optimized search engines from custom scripts. Among a plethora of annotation packages on Bioconductor ( www.bioconductor.org) and CRAN ( www.cran-project.org), *myvariant* ^3^, *biomaRt* ^4^, *rentrez* ^5^ can retrieve information about annotated SNPs. However, even rich outputs of these packages lack information about LD, eQTL, AF and haplotype blocks. We present an R package, *haploR*, which allows querying HaploReg and RegulomeDB web-based tools from R environment. The package connects to the web site, queries the database, and downloads results into a data frame. HaploR can easily be included in bioinformatics pipelines, which will facilitate search for SNP -phenotype associations.

We present an R package, *haploR*, which allows querying HaploReg and RegulomeDB web-based tools from R environment. The package connects to the web site, queries the database and downloads results into a data frame. *haploR* can easily be included in bioinformatics pipelines, which will facilitate search for SNP - phenotype associations.

## Methods

### Implementation

*haploR* relies on HTTP methods POST and GET to query and download the content of web pages. Functions queryHaploreg(...) and queryRegulome(...) are designed to query the HaploReg ( http://archive.broadinstitute.org/mammals/haploreg/haploreg.php) and RegulomeDB ( http://www.regulomedb.org/), respectively. The structure of the retrieved data is described on the package website and corresponding vignette.

### Operation

The package is cross-platform (Windows, macOS and Linux), without any specific computer hardware requirements. A standard computer with the most-recent version of R will handle most applications of the *haploR* package. Installation instructions and a list of prerequisites are provided on the package web page.

## Use cases

### Querying HaploReg

To query HaploReg, the user needs to call queryHaploreg(query, file, study, ...). This function can accept three different inputs: (1) a vector of SNPs (query); (2) a text file ( file); or (3) a study ( study) that can be obtained from HaploReg using getHaploregStudyList(). Parameters of these functions are directly linked to options provided at the HaploReg web page and described in the package user manual. Examples below show usage of a vector of SNPs. For other examples please refer to the package vignette.



                        library(haploR)
x <- queryHaploreg(query=c("rs10048158","rs4791078"))
                    


Here parameter query represents a vector of SNPs identified with rs-IDs.

### Querying RegulomeDB

The RegulomeDB project also allows exploration of properties of SNPs and presents results in different formats: (1) plain text (vector of rs-ID) (2) BED and (3) GFF formats. The function queryRegulome(query, ...) is used to query the RegulomeDB:



                        x <- queryRegulome(query=c("rs4791078","rs10048158"))
                    


Here the query is a vector of rs-IDs. The output is similar to that used in the queryHaploreg function in terms of the type of information retrieved, but specific to the RegulomeDB output. For detailed format explanations refer to the RegulomeDB web site.

## Conclusion and future work

*haploR* can be easily included to bioinformatics pipeline to streamline the process and reduce the analysis time. Its advantages over the original databases include: shorter retrieval time, the ability to present results in a user-friendly form (allowing for a more streamlined workflow,) and convenient use of needed information in reports, presentations and publications. We plan to add other tools, such as Regulatory Elements ( http://dnase.genome.duke.edu/index.php), which provides the data from DNaseI hypersensitivity and microarray experiments performed in 6. Understanding the factors modulating gene expression and protein yield across individuals can be beneficial. Cell types may help discover novel mechanisms of genetic associations.

## Software availability

Tool available from: https://cran.r-project.org/package=haploR

Source code available from: https://github.com/izhbannikov/haploR

Archived source as at time of publication: https://cran.r-project.org/src/contrib/haploR_1.4.4.tar.gz, doi: https://doi.org/10.5281/zenodo.570956

License: GPL-3

## Data availability

The data referenced by this article are under copyright with the following copyright statement: Copyright: © 2017 Zhbannikov IY et al.

Data associated with the article are available under the terms of the Creative Commons Zero "No rights reserved" data waiver (CC0 1.0 Public domain dedication). 

The example script and output files for the package are available at: https://doi.org/10.5281/zenodo.570960

## Supplementary material

***haploR*-vignette. Using haploR, an R package for querying HaploReg and RegulomeDB.** This file includes a description of post-GWAS analysis and the unique contribution of the haploR to it. It also includes an example of a typical analysis workflow using haploR. There is also a description of the post-GWAS web databases (HaploReg, RegulomeDB) used in the package with comprehensive examples of usage. This file also describes the data structures used in haploR.
