# Supplementary material for: haploR: an R package for querying web-based annotation tools
Source: F1000Res. 2017 May 15;6:97. Originally published 2017 Feb 1. [Version 2] doi: 10.12688/f1000research.10742.2 (PMC5461892; doi:10.12688/f1000research.10742.2)
Supplement: Supplementary file 1 [file f1000research-6-12496-s0000.tgz › c102caad-f2d8-4643-b379-9217c7a685b3.pdf]

Using haploR, an R package for querying HaploReg and RegulomeDB

Ilya Y. Zhbannikov

Thu May 4 01:46:36 2017

Overview

HaploReg (http://archive.broadinstitute.org/mammals/haploreg/haploreg.php) and RegulomeDB (http://www.regulomedb.org) are web-based tools that extract biological information such as eQTL, LD, motifs, etc. from large genomic projects such as ENCODE, the 1000 Genomes Project, Roadmap Epigenomics Project and others. This is sometimes called "post stage GWAS" analysis.

The R-package *haploR* was developed to query those tools (HaploReg and RegulomeDB) directly from *R* in order to facilitate high-throughput genomic data analysis. Below we provide several examples that show how to work with this package.

Note: you must have a stable Internet connection to use this package.

Contact: ilya.zhbannikov@duke.edu for questions of usage the *haploR* or any other issues.

Motivation and typical analysis workflow

This package was inspired by the fact that many web-based post stage GWAS databases do not have Application Programing Interface (API) and, therefore, do not allow users to query them remotedly from R environment. In our research we used HaploReg and RegulomeDB web databases. These very useful web databases show information about linkage disequilibrium of query variants and variants which are in LD with them, expression quantitative trait loci (eQTL), motifs changed and other useful information. However, it is not easy to include this information into streamlined workflow since those tools also not offer API.

We developed a custom analysis pipeline which prepares data, performs genome-wide association (GWA) analysis and presents results in a user-friendly format. Results include a list of genetic variants (also known as 'SNP' or single nucleotide polymorphism), their corresponding *p*-values, phenotypes (traits) tested and other meta-information such as LD, alternative allele, minor allele frequency, motifs changed, etc. Of course, we could go throught the list of SNPs having genome-wide significant *p*-values (1e-8) and submit each of those SNPs to HaploReg or RegulomeDB manually, one-by-one, but it is time-consuming process and will not be fully automatic (which ruins one of the pipeline's paradigms). This is especially difficult if the web site does not offer downloading results.

Therefore, we developed *haploR*, a user-friendly R package that connects to HaploReg or RegulomeDB from R environment with methods POST and GET and downloads results in a suitable R format. This package siginificantly saved our time in developing reporting system for our internal genomic analysis pipeline and now we would like to present *haploR* to the research community.

Example of typical analysis workflow is shown below.

- Data preprocessing stage usually consists of basic cleaning operations (sex inconsistencies check, filtering by MAF, missing genotype rate), format conversion, population stratification, creating temporaty files, etc.
- Genome-wide association study (GWAS). This Includes testing hyphotesis on correlation between phenotype and genotype. Usually in form of linear or logistic regression but can be quite sophisticated especially for rare variants.

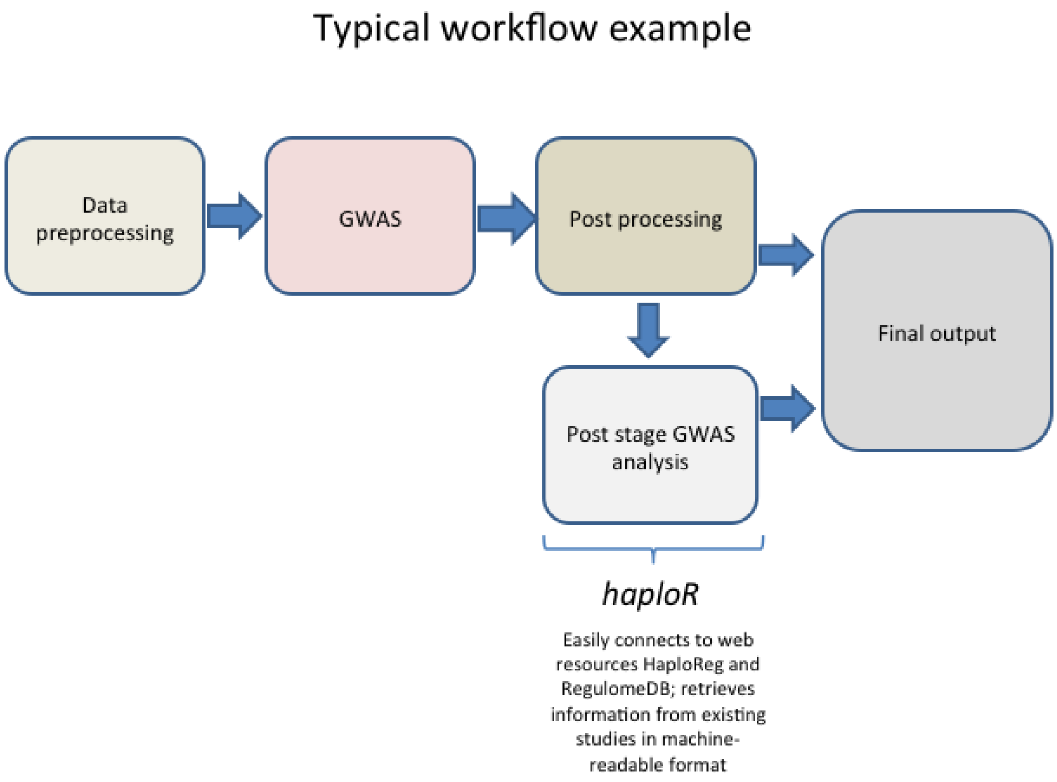

Figure 1: Typical analysis workflow

- Postprocessing usually includes summarizing results in tables, creating graphics (Manhattan plot, QQ-plot), sometimes filtering by significance threshold (usually 1E-8), removing temporary files, etc.
- Post stage GWAS analysis: connect GWAS findings with existing studies and gather information about linked SNPs, chromatin state, protein binding annotation, sequence conservation across mammals, effects on regulatory motifs and on gene expression, etc. This helps researchers to more understand functions and test additional hyphotesis of found genome-wide significant SNPs. At this stage *haploR* is especially useful because it provides a convenient R interface to mining web databases. This, in turn, streamlines analysis workflow and therefore significantly reduces analysis time. Previously researchers had to do it manually after analysis by exploring available web resources, checking each SNP of interest and downloading results (which is especially painful if website does not have a download option). With *haploR* such post-GWAS information are easily retrieved, shared and appeared in the final output tables at the end of analysis. This will save researchers' time.

Installation of *haploR* package

In order to install the *haploR* package, the user must first install R (https://www.r-project.org). After that, *haploR* can be installed either:

- From CRAN (stable version):

```
install.packages("haploR", dependencies = TRUE)
```

- Or from the package web page (developing version):

```
devtools::install_github("izhbannikov/haplor")
```

haploR depends on the following packages:

- *httr*, version 1.2.1 or later.
- *XML*, version version 3.98-1.6 or later.
- *tibble*, version 1.3.0 or later.
- *RUnit*, version 0.4.31 or later.

Examples of usage

Querying HaploReg

Function

```
queryHaploreg(query = NULL, file = NULL, study = NULL, ldThresh = 0.8,
  ldPop = "EUR", epi = "vanilla", cons = "siphy", genetypes = "gencode",
  url = "http://archive.broadinstitute.org/mammals/haploreg/haploreg.php",
  timeout = 10, encoding = "UTF-8", verbose = FALSE)
```

queries HaploReg web-based tool and returns results.

Arguments

- *query*: Query (a vector of rsIDs).
- *file*: A text file (one refSNP ID per line).
- *study*: A particular study. See function getHaploRegStudyList(…). Default: `NULL`.
- *ldThresh*: LD threshold, r2 (select NA to show only query variants). Default: `0.8`.
- *ldPop*: 1000G Phase 1 population for LD calculation. Can be: `AFR` (Africa), `AMR` (America), `ASN` (Asia). Default: `EUR` (Europe).

- *epi*: Source for epigenomes. Possible values: `vanilla` for ChromHMM (Core 15-state model); `imputed` for ChromHMM (25-state model using 12 imputed marks); `methyl` for H3K4me1/H3K4me3 peaks; `acetyl` for H3K27ac/H3K9ac peaks. Default: `vanilla`.
- *cons*: Mammalian conservation algorithm. Possible values: `gerp` for GERP (http://mendel.stanford.edu/SidowLab/downloads/gerp/), `siphy` for SiPhy-omega, `both` for both. Default: siphy.
- *genetypes*: Show position relative to. Possible values: `gencode` for Gencode genes; `refseq` for RefSeq genes; `both` for both. Default: `gencode`.
- *url*: HaploReg url address. Default: http://archive.broadinstitute.org/mammals/haploreg/haploreg.php
- *timeout*: A timeout parameter for curl. Default: `10`
- *encoding*: Set the encoding for correct retrieval web-page content. Default: `UTF-8`
- *verbose*: Verbosing output. Default: `FALSE`.

Output

A data frame (table) wrapped into a *tibble* object contains data extracted from HaploReg site. The colums (33 in total at the time of writing this vignette) are specific to HaploReg output. Below we describe the columns:

- *chr*: Chromosome, type: numeric
- *pos_hg38*: Position on the human genome, type: numeric.
- *r2*: Linkage disequilibrium. Type: numeric.
- D': Linkage disequilibrium, alternative definition. Type: numeric.
- *is_query_snp*: Indicator shows query SNP, 0 - not query SNP, 1 - query SNP. Type: numeric.
- *rsID*: refSNP ID. Type: character.
- *ref*: Reference allele. Type: character.
- *alt*: Alternative allele. Type: character.
- *AFR*: *r2* calculated for Africa. Type: numeric.
- *AMR*: *r2* calculated for America. Type: numeric.
- *ASN*: *r2* calculated for Asia. Type: numeric.
- *EUR*: *r2* calculated for Europe. Type: numeric.
- *GERP_cons*: GERP scores. Type: numeric.
- *SiPhy_cons*: SiPhy scores. Type: numeric.
- *Chromatin_States*: Chromatin states: reference epigenome identifiers (EID) of chromatin-associated proteins and histone modifications in that region. Type: character.
- *Chromatin_States_Imputed*: Chromatin states based on imputed data. Type: character.
- *Chromatin_Marks*: Chromatin marks Type: character.
- *DNAse*: DNAse. Type: chrarcter.
- *Proteins*: A list of protein names. Type: character.
- *eQTL*: Expression Quantitative Trait Loci. Type: character.
- *gwas*: GWAS study name. Type: character.
- *grasp*: GRASP study name: character.
- *Motifs*: Motif names. Type: character.
- *GENCODE_id*: GENCODE transcript ID. Type: character.
- *GENCODE_name*: GENCODE gene name. Type: character.
- *GENCODE_direction*: GENCODE direction (transcription toward 3' or 5' end of the DNA sequence). Type: numeric.
- *GENCODE_distance*: GENCODE distance. Type: numeric.
- *RefSeq_id*: NCBI Reference Sequence Accession number. Type: character.
- *RefSeq_name*: NCBI Reference Sequence name. Type: character.
- *RefSeq_direction*: NCBI Reference Sequence direction (transcription toward 3' or 5' end of the DNA sequence). Type: numeric.
- *RefSeq_distance*: NCBI Reference Sequence distance. Type: numeric.
- *dbSNP_functional_annotation* Annotated proteins associated with the SNP. Type: numeric.
- *query_snp_rsid*: Query SNP rs ID. Type: character.

Number of rows is not constant, at least equal or more than the number of query SNPs, and depends on *r2* parameter choosen in a query (default 0.8). This means that the program outputs not only query SNPs, but also those SNPs that have *r2* ≥ 0.8 with the query SNPs.

One or several genetic variants

```
library(haploR)
x <- queryHaploreg(query=c("rs10048158","rs4791078"))
x
```

```
## # A tibble: 33 x 33
##      chr pos_hg38    r2  `D'` is_query_snp       rsID   ref   alt   AFR
##    <dbl>    <dbl> <dbl> <dbl>        <dbl>      <chr> <chr> <chr> <dbl>
## 1     17 66213160  0.82  0.93            0  rs4790914     C     G  0.84
## 2     17 66213422  0.82  0.93            0  rs4791079     T     G  0.85
## 3     17 66213896  0.82  0.93            0  rs4791078     A     C  0.84
## 4     17 66214285  0.83  0.93            0  rs1971682     G     C  0.86
## 5     17 66216124  0.83  0.93            0  rs4366742     T     C  0.93
## 6     17 66219453  0.83  0.93            0  rs2215415     G     A  0.91
## 7     17 66220526  0.83  0.93            0  rs3744317     G     A  0.93
## 8     17 66227121  0.83  0.94            0  rs8178827     C     T  0.90
## 9     17 66230111  0.83  0.93            0 rs71160546    GA     G  0.87
## 10    17 66231972  0.82  0.99            0 rs11079645     G     T  0.88
## # ... with 23 more rows, and 24 more variables: AMR <dbl>, ASN <dbl>,
## #   EUR <dbl>, GERP_cons <dbl>, SiPhy_cons <dbl>, Chromatin_States <chr>,
## #   Chromatin_States_Imputed <chr>, Chromatin_Marks <chr>, DNAse <chr>,
## #   Proteins <chr>, eQTL <chr>, gwas <chr>, grasp <chr>, Motifs <chr>,
## #   GENCODE_id <chr>, GENCODE_name <chr>, GENCODE_direction <dbl>,
## #   GENCODE_distance <dbl>, RefSeq_id <chr>, RefSeq_name <chr>,
## #   RefSeq_direction <dbl>, RefSeq_distance <dbl>,
## #   dbSNP_functional_annotation <chr>, query_snp_rsid <chr>
```

Here *query* is a vector with names of genetic variants.

We then can create a subset from the results, for example, to choose only SNPs with r2 > 0.9:

```
subset.high.LD <- x[x$r2 > 0.9, c("rsID", "r2", "chr", "pos_hg38", "is_query_snp", "ref", "alt")]
subset.high.LD
```

```
## # A tibble: 13 x 7
##          rsID    r2   chr pos_hg38 is_query_snp   ref   alt
##         <chr> <dbl> <dbl>    <dbl>        <dbl> <chr> <chr>
## 1  rs10048158  1.00    17 66240200            1     T     C
## 2   rs9895261  1.00    17 66244318            0     A     G
## 3  rs12603947  0.99    17 66248387            0     T     C
## 4   rs7342920  0.99    17 66248527            0     T     G
## 5   rs4790914  1.00    17 66213160            0     C     G
## 6   rs4791079  1.00    17 66213422            0     T     G
## 7   rs4791078  1.00    17 66213896            1     A     C
## 8   rs1971682  0.98    17 66214285            0     G     C
## 9   rs4366742  0.99    17 66216124            0     T     C
## 10  rs2215415  0.99    17 66219453            0     G     A
## 11  rs3744317  0.99    17 66220526            0     G     A
## 12  rs8178827  0.96    17 66227121            0     C     T
## 13 rs71160546  0.94    17 66230111            0    GA     G
```

We can then save the *subset.high.LD* into an Excel workbook:

```
require(openxlsx)
write.xlsx(x=subset.high.LD, file="subset.high.LD.xlsx")
```

This was an example of gathering post-gwas information directly from the online tool. *haploR* has an additional advantage because it downloads the full information for query retrieved by HaploReg. For example, if you go online and submit these two SNPs to HaploReg (http://archive.broadinstitute.org/mammals/haploreg/haploreg.php), you will see that some cells of columns "Motifs changed" and "Selected eQTL hits" are hidded (only number of hits are given). *haploR* retrives this information in a form of a data frame which can be saved into Excel file.

```
x[, c("Motifs", "rsID")]
```

```
## # A tibble: 33 x 2
##                                                                              Motifs
##                                                                               <chr>
## 1  AP-4_3;Ascl2;E2A_5;Foxa_disc3;HEN1_2;LBP-1_2;NRSF_disc4;NRSF_disc8;NRSF_kno
## 2                                  Pou5f1_disc1;RFX5_known1;Sox_4;TATA_disc7
## 3                                                          RFX5_known5;Zbtb3
## 4                   AP-1_disc1;HEN1_1;Maf_disc2;NR4A_known1;RAR;RXRA_known3;T3R
## 5                                                                     Pdx1_2
## 6                                               AP-1_disc1;HEY1_disc1;TATA_disc2
## 7                                                                          .
## 8                                                ATF3_disc1;LXR_2;SREBP_known4
## 9                                                 Evi-1_3;Irf_disc3;STAT_disc3
## 10                                                             GR_disc4;SZF1-1
## # ... with 23 more rows, and 1 more variables: rsID <chr>
```

```
x[, c("eQTL", "rsID")]
```

```
## # A tibble: 33 x 2
##                                                                                eQTL
##                                                                               <chr>
## 1                GTEx2015_v6,Heart_Left_Ventricle,PRKCA,3.25964766049438e-10
## 2  GTEx2015_v6,Heart_Left_Ventricle,PRKCA,2.87827072933431e-10;Koopman2014,Hea
## 3                GTEx2015_v6,Heart_Left_Ventricle,PRKCA,2.87827072933431e-10
## 4                GTEx2015_v6,Heart_Left_Ventricle,PRKCA,5.94596439339797e-11
## 5                GTEx2015_v6,Heart_Left_Ventricle,PRKCA,6.83955923561212e-11
## 6                GTEx2015_v6,Heart_Left_Ventricle,PRKCA,6.80544182605399e-11
## 7  GTEx2015_v6,Heart_Left_Ventricle,PRKCA,6.80544182605399e-11;Koopman2014,Hea
## 8                GTEx2015_v6,Heart_Left_Ventricle,PRKCA,2.44658806733437e-10
## 9                GTEx2015_v6,Heart_Left_Ventricle,PRKCA,2.09660151875432e-10
## 10               GTEx2015_v6,Heart_Left_Ventricle,PRKCA,6.18988623085424e-12
## # ... with 23 more rows, and 1 more variables: rsID <chr>
```

Uploading file with variants

If you have a file with your SNPs you would like to analyze, you can supply it as an input as follows:

```
library(haploR)
x <- queryHaploreg(file=system.file("extdata/snps.txt", package = "haploR"))
x
```

```
## # A tibble: 33 x 33
##      chr pos_hg38    r2  `D'` is_query_snp       rsID   ref   alt   AFR
##    <dbl>    <dbl> <dbl> <dbl>        <dbl>      <chr> <chr> <chr> <dbl>
```

```
## 1     17 66213160  0.82  0.93            0  rs4790914     C     G  0.84
## 2     17 66213422  0.82  0.93            0  rs4791079     T     G  0.85
## 3     17 66213896  0.82  0.93            0  rs4791078     A     C  0.84
## 4     17 66214285  0.83  0.93            0  rs1971682     G     C  0.86
## 5     17 66216124  0.83  0.93            0  rs4366742     T     C  0.93
## 6     17 66219453  0.83  0.93            0  rs2215415     G     A  0.91
## 7     17 66220526  0.83  0.93            0  rs3744317     G     A  0.93
## 8     17 66227121  0.83  0.94            0  rs8178827     C     T  0.90
## 9     17 66230111  0.83  0.93            0 rs71160546    GA     G  0.87
## 10    17 66231972  0.82  0.99            0 rs11079645     G     T  0.88
## # ... with 23 more rows, and 24 more variables: AMR <dbl>, ASN <dbl>,
## #   EUR <dbl>, GERP_cons <dbl>, SiPhy_cons <dbl>, Chromatin_States <chr>,
## #   Chromatin_States_Imputed <chr>, Chromatin_Marks <chr>, DNAse <chr>,
## #   Proteins <chr>, eQTL <chr>, gwas <chr>, grasp <chr>, Motifs <chr>,
## #   GENCODE_id <chr>, GENCODE_name <chr>, GENCODE_direction <dbl>,
## #   GENCODE_distance <dbl>, RefSeq_id <chr>, RefSeq_name <chr>,
## #   RefSeq_direction <dbl>, RefSeq_distance <dbl>,
## #   dbSNP_functional_annotation <chr>, query_snp_rsid <chr>
```

File "snps.txt" is a text file which contains one rs-ID per line:

```
rs10048158
rs4791078
```

Using existing studies

Sometimes one would like to explore results from already performed study. In this case you should first explore the existing studies from HaploReg web site (http://archive.broadinstitute.org/mammals/haploreg/haploreg.php) and then use one of them as an input parameter. See example below:

```
library(haploR)
# Getting a list of existing studies:
studies <- getStudyList()
# Let us look at the first element:
studies[[1]]
```

```
## $name
## [1] "<ce><b2>2-Glycoprotein I (<ce><b2>2-GPI) plasma levels (Athanasiadis G, 2013, 9 SNPs)"
##
## $id
## [1] "1756"
```

```
# Let us look at the second element:
studies[[2]]
```

```
## $name
## [1] "5-HTT brain serotonin transporter levels (Liu X, 2011, 1 SNP)"
##
## $id
## [1] "2362"
```

```
# Query Hploreg to explore results from
# this study:
x <- queryHaploreg(study=studies[[1]])
x
```

```
## # A tibble: 117 x 33
##      chr pos_hg38    r2  `D'` is_query_snp       rsID   ref   alt   AFR
```

```
##     <dbl>    <dbl> <dbl> <dbl>        <dbl>      <chr> <chr> <chr> <dbl>
## 1      11 34524785  0.97  1.00            0   rs836138     C     A  0.34
## 2      11 34524788  0.87  0.97            0 rs11032744     C     T  0.04
## 3      11 34526877  1.00  1.00            0   rs836137     A     G  0.37
## 4      11 34527359  1.00  1.00            0   rs836135     G     A  0.36
## 5      11 34527815  1.00  1.00            0   rs704727     T     A  0.16
## 6      11 34530979  0.96  0.99            0   rs836133     C     T  0.16
## 7      11 34531545  0.90  1.00            0 rs77003093     C     T  0.01
## 8      11 34533644  1.00  1.00            1   rs836132     G     A  0.16
## 9      11 34534390  1.00  1.00            0   rs836131     C     T  0.16
## 10     11 34535548  1.00  1.00            0   rs836130     G     T  0.36
## # ... with 107 more rows, and 24 more variables: AMR <dbl>, ASN <dbl>,
## #   EUR <dbl>, GERP_cons <dbl>, SiPhy_cons <dbl>, Chromatin_States <chr>,
## #   Chromatin_States_Imputed <chr>, Chromatin_Marks <chr>, DNAse <chr>,
## #   Proteins <chr>, eQTL <chr>, gwas <chr>, grasp <chr>, Motifs <chr>,
## #   GENCODE_id <chr>, GENCODE_name <chr>, GENCODE_direction <dbl>,
## #   GENCODE_distance <dbl>, RefSeq_id <chr>, RefSeq_name <chr>,
## #   RefSeq_direction <dbl>, RefSeq_distance <dbl>,
## #   dbSNP_functional_annotation <chr>, query_snp_rsid <chr>
```

Querying RegulomeDB

To query RegulomeDB use this function:

```
queryRegulome(query = NULL,
              format = "full",
              url = "http://www.regulomedb.org/results",
              timeout = 10,
              check_bad_snps = TRUE,
              verbose = FALSE)
```

This function queries RegulomeDB http://www.regulomedb.org web-based tool and returns results in a named list.

Arguments

- *query*: Query (a vector of rsIDs).
- *format*: An output format. Only 'full' is currently supported. See `http://www.regulomedb.org/results`.
- *url*: Regulome url address. Default: `http://www.regulomedb.org/results`
- *timeout*: A 'timeout' parameter for 'curl'. Default: 10.
- *check_bad_snps*: Checks if all query SNPs are annotated (i.e. presented in the Regulome Database). Default: 'TRUE'
- *verbose*: Verbosing output. Default: FALSE.

Output

A list of two: (1) a data frame (res.table) wrapped to a *tibble* object and (2) a list of bad SNP IDs (bad.snp.id). Bad SNP ID are those IDs that were not found in 1000 Genomes Phase 1 data and, therefore, in RegulomeDB.

Columns in a data frame (res.table):

- *#chromosome*: Chromosome. Type: character.
- *coordinate*: Position. Type: numeric.
- *rsid*: RefSeq SNP ID. Type: character.
- *hits*: Contains information about chromatin structure: method and cell type. Type: character.

- *score*: Internal RegulomeDB score. See http://www.regulomedb.org/help#score. Type: numeric.

Number of rows is equal or less (in case if not all SNPs were found in RegulomeDB database) to number of query SNPs.

All the information retreived from RegulomeDB, except *hits*, are also presented in HaploReg output (`queryHaploReg(...)`).

Example

```
library(haploR)
x <- queryRegulome(c("rs4791078","rs10048158"))
x$res.table
```

```
## # A tibble: 2 x 5
##   `#chromosome` coordinate       rsid
##           <chr>      <dbl>      <chr>
## 1         chr17   64236317 rs10048158
## 2         chr17   64210013  rs4791078
## # ... with 2 more variables: hits <chr>, score <dbl>
```

```
x$bad.snp.id
```

```
## # A tibble: 0 x 1
## # ... with 1 variables: rsID <chr>
```

Session information

```
sessionInfo()
```

```
## R Under development (unstable) (2017-03-04 r72303)
## Platform: x86_64-apple-darwin13.4.0 (64-bit)
## Running under: macOS Sierra 10.12.4
##
## Matrix products: default
## BLAS: /Library/Frameworks/R.framework/Versions/3.4/Resources/lib/libRblas.0.dylib
## LAPACK: /Library/Frameworks/R.framework/Versions/3.4/Resources/lib/libRlapack.dylib
##
## locale:
## [1] C
##
## attached base packages:
## [1] stats     graphics  grDevices utils     datasets  methods   base
##
## other attached packages:
## [1] haploR_1.4.4
##
## loaded via a namespace (and not attached):
##  [1] Rcpp_0.12.10    XML_3.98-1.6    digest_0.6.12   rprojroot_1.2
##  [5] mime_0.5        R6_2.2.0        backports_1.0.5 magrittr_1.5
##  [9] evaluate_0.10   httr_1.2.1      stringi_1.1.5   curl_2.4
## [13] RUnit_0.4.31    rmarkdown_1.4   tools_3.4.0     stringr_1.2.0
## [17] yaml_2.1.14     compiler_3.4.0  htmltools_0.3.5 knitr_1.15.1
## [21] tibble_1.3.0
```
