# Supplementary material for: haploR: an R package for querying web-based annotation tools
Source: F1000Res. 2017 May 15;6:97. Originally published 2017 Feb 1. [Version 2] doi: 10.12688/f1000research.10742.2 (PMC5461892; doi:10.12688/f1000research.10742.2)
Supplement: Peer review history

# Peer review: Referee response for version 2

**DOI:** 10.5256/f1000research.12496.r22714

**Published:** 2017-07-03

**Authors:** Claudia Vitolo, Estibaliz Gascon, Fatima Pillosu

**Affiliations:** 1 European Centre for Medium-Range Weather Forecasts, Reading, UK; 2 European Centre for Medium-Range Weather Forecasts, Reading, UK, Reading, UK

The authors have addressed my concerns.

 I only have few minor comments:

- There is a repetition in the last part of the introduction (The package connects to the web site...)
- In the text you mention the 'package website'. If I understand well, this is actually the package repository on GitHub, right? Just make that clearer in the text.
- R CMD check shows that there is a mismatch between documentation and code for function queryRegulome(), please fix argument 'timeout' (default value in Code: 100 while in Docs: 10)

Many thanks to the author for their hard work on this revision.

We have read this submission. We believe that we have an appropriate level of expertise to confirm that it is of an acceptable scientific standard.

# Peer review: Referee response for version 2

**DOI:** 10.5256/f1000research.12496.r22712

**Published:** 2017-05-31

**Authors:** Garrett M. Dancik

**Affiliations:** 1 Department of Computer Science, Eastern Connecticut State University, Willimantic, CT, USA

The authors have addressed my concerns.

I have read this submission. I believe that I have an appropriate level of expertise to confirm that it is of an acceptable scientific standard.

# Peer review: Referee response for version 2

**DOI:** 10.5256/f1000research.12496.r22713

**Published:** 2017-05-30

**Authors:** Stephanie M. Gogarten

**Affiliations:** 1 Department of Biostatistics, University of Washington, Seattle, WA, USA

The authors have addressed my concerns. My only additional comment is that the last two sentences of the Introduction are now redundant with the previous paragraph and should be deleted.

I have read this submission. I believe that I have an appropriate level of expertise to confirm that it is of an acceptable scientific standard.

# Peer review: Referee response for version 1

**DOI:** 10.5256/f1000research.11583.r20081

**Published:** 2017-03-03

**Authors:** Stephanie M. Gogarten

**Affiliations:** 1 Department of Biostatistics, University of Washington, Seattle, WA, USA

This paper describes an R-package, *haploR*, which queries bionformatics databases. The benefit of the package is an ability to incorporate these queries into workflows in R, rather than using a web interface.

 The *haploR* package seems useful, but the paper is lacking sufficient detail in several areas. 

1. The Bioconductor project (bioconductor.org) contains a wealth of resources for querying various sources of annotation from R. The paper should discuss how the *haploR* package provides features that are not available in existing resources.
2. The types of information available in HaploReg and RegulomeDB are not well described. Why were these particular resources selected for this package and how do they differ from each other?
3. The "future work" section mentions adding other web tools to the package in the future. What additional information will be provided by those tools and how were they selected for inclusion in the package?

I was able to install the R-package and follow the examples given in the vignette. However, these examples would benefit from more explanation.

1. In the HaploReg example, querying the database with two rs IDs returns results for many additional rs IDs. Why is this?
2. Why is the first element returned by getStudyList() blank?

In summary, the authors have provided a potentially useful R-package, but they need to include more explanation of how this package will benefit the bioinformatics community.

I have read this submission. I believe that I have an appropriate level of expertise to confirm that it is of an acceptable scientific standard, however I have significant reservations, as outlined above.

# Response

**Published:** 2017-05-04

**Authors:** Ilya Zhbannikov

**Affiliations:** Duke University, USA

We thank the reviewer for careful reading of our paper and constructive remarks. We believe that the comments have identified important areas which required improvement. After completion of the suggested edits, the revised paper has benefited from an improvement in the overall presentation and clarity. Reviewer comments/suggestions (RC) are in italics font; our responses (AR) are in regular, black font.

**RC1:**

*The Bioconductor project (bioconductor.org) contains a wealth of resources for querying various sources of annotation from R. The paper should discuss how the haploR package provides features that are not available in existing resources.*

**AR1:**

We wanted to automatically retrieve the information about annotated genetic variants listed as an output of our custom genomic pipeline. We decided to find an R package that would be able to do this rather than download very large annotation files from different projects in order to query them locally. Among a plethora of annotation packages from Bioconductor and CRAN ( *annotate*, *mygene*, *ensembldb*, *biomaRt*, *myvariant*, *rsnps*, *rentrez*), only *myvariant*, *biomaRt*, *rentrez* could potentially serve our needs. However, even the rich outputs of *myvariant*, *biomaRt* and *rentrez* did not contain ready-to use information about LD, sequence conservation across mammals, the effect of SNPs on regulatory motifs, and the effect of SNPs on expression from eQTL studies. In the revised version of our paper we briefly (due to limited size) emphasized the advantages of haploR. Please see introductory section.

**RC2:**

*The types of information available in HaploReg and RegulomeDB are not well described. Why were these particular resources selected for this package and how do they differ from each other?*

**AC2:**

HaploReg is a web resource for exploring annotations of genetically linked variants (i.e. variants in haplotype blocks). The particular advantage of HaploReg is that it allows explorations the effects of SNPs on expression from eQTL studies. It also outputs genetically linked (to the query) SNPs, therefore we can discover effects of correlations. RegulomeDB is a resource that shows annotated SNPs with known and predicted regulatory elements in the intergenic regions of the human genome. Data mostly come from publicly available datasets (GEO, ENCODE, etc.). Both HaploReg and RegulomeDB were chosen as convenient tools for exploring effects of eQTL and determining close-related variants. We added description of HaploReg and RegulomeDB output data to the package vignette (please see Overview section).

**RC3:**

*The "future work" section mentions adding other web tools to the package in the future.  What additional information will be provided by those tools and how were they selected for inclusion in the package?*

**AC3:**

We think that including additional resources on regulatory factors is beneficial since such factors can modulate gene expression and protein yield distinctly across individuals and cell types. This can help us to discover novel mechanisms of genetic associations.

**RC4:**

*I was able to install the R-package and follow the examples given in the vignette. However, these examples would benefit from more explanation. In the HaploReg example, querying the database with two rs IDs returns results for many additional rs IDs. Why is this?*

**AC4:**

This happened because HaploReg returns information about query SNPs and also information about those SNPs, which are in LD equal or higher than some pre-defined threshold (0.8 by default).

**RC5:**

*Why is the first element returned by getStudyList() blank?*

**AC5:**

This was because we used a study list returned by Haploreg 'as is' where the first element was blank. It is fixed in version 1.4.4 of the package (blanks were removed).

# Peer review: Referee response for version 1

**DOI:** 10.5256/f1000research.11583.r19826

**Published:** 2017-02-23

**Authors:** Claudia Vitolo, Estibaliz Gascon, Fatima Pillosu

**Affiliations:** 1 European Centre for Medium-Range Weather Forecasts, Reading, UK; 2 European Centre for Medium-Range Weather Forecasts, Reading, UK, Reading, UK

This papers describes the implementation of the *haploR* R-package which is used to retrieve information from web-based genome annotation tools. This R-package aims to simplify the reproducibility of bioinformatics pipe lines.

 Overall, we think the structure of the paper and the aim of the project are inline with the journal’s guidelines. The *haploR *package seems a valuable open source tool for bioinformaticians and R users as it facilitates data retrieval from web-based databases (such as HaploReg and RegulomeDB) and makes the scientific workflow more reproducible. We also appreciate the intention to keep improving the package by extending the list of supported databases.

 We mostly work on climate science and have a limited understanding of bioinformatics. However, we use R extensively and we decided to review this work from a generic R-user perspective. We focused our review on this paper and source code, we considered user manual and the vignette out of the scope of this review.

 In our opinion, this paper deserves publication but requires some further work. We decided to approve it with reservations because we noticed some ambiguities in the paper that need to be clarified. We also suggest small changes to the code that could make the functions in the package less error-prone and more future proof. Our specific comments are listed below.

**Major comments**

1. INTRODUCTION
   
  - We think the introduction is rather vague. There are several sentences such as “in a number of situations” or “in a certain format” which are too vague and require further explanations. For example, instead of saying “in a certain format”, the authors could explicitly mention the formats that they are referring to (e.g. csv, json, etc). Again, in the second sentence of the third paragraph “... saving the results of such analyses in different file formats ...” the authors should again specify what the different file formats are.
  - Just before the fourth paragraph, the authors should mention if this package could be added to one of the CRAN Task Views ( https://cran.r-project.org/web/views/) and whether there are other packages with similar goals. If there are other related packages, it would be interesting to mention whether the data could be combined.  
2. METHODS
   
  - The second sentence of the sub-section Implementation says “Functions….are designed to obtain data from the resources HaploReg...and RegulomeDB….”. Here, it is important to describe the structure of the retrieved data.
  - We appreciate that most bioinformaticians are familiar with web-based databases such as HaploReg and RegulomeDB. However, a student might want to use this tool and having a more detailed description of these web databases would be useful to get started. Please, also consider commenting on the use and interpretation of the retrieved information, for example plotting a subset of the full dataset.
  - The Operation section should include clear instructions for the installation and a complete description of package dependencies, including versions of the dependent packages.
3. USE CASES
   
  - This section is rather vague. The authors should clearly describe all the input arguments of the functions, as well as the expected results.
  - Querying HaploReg - Input vector of SNPs
    - When writing example code, it is considered good practice to assign the result of a command to an object, e.g. x <- queryHaploreg(query=c("rs10048158","rs4791078")). Please consider making this change throughout the paper.
    - When we run the command x <- queryHaploreg(query=c("rs10048158","rs4791078")) we get the following message: “No encoding supplied: defaulting to UTF-8”. Consider changing the encoding or removing non-Ascii characters from the table before outputting.
    - After retrieving the data, please describe the structure of the retrieved object. In particular you should mention the expected number of columns and rows as well as the name and type of variables (the authors might find the str() function useful).
    - We tried to print the object, the result filled the screen and was unreadable. We suggest to convert the dataframe into a tibble table (see tibble package) to generate a more readable printed output.
    - We checked the structure of the retrieved objects and the data types are all characters. Some of the columns clearly contain numeric variables (e.g. r2, D , ARF…). We suggest to convert there columns from character to numeric before outputting. This conversion is important because users might incur into errors when generating basic statistics. For instance, running x <- queryHaploreg(query=c("rs10048158","rs4791078")); quantile(x$AFR) generates the following error message: “Error in (1 - h) * qs[i] : non-numeric argument to binary operator”.
  - Querying HaploReg - Input text file with SNPs: This example is reproducible but the authors do not specify how the "extdata/snps.txt” is structured. We suggest to write something like “the text file should list the rs-IDs in one column, with one rs-ID per row”. 
  - Querying HaploReg - Using a particular study: When we extracted the list of studies, we noticed that we cannot subset it using names. Subsetting using indices is prone to errors because the list of studies could increase over time and their order could change.  
  - Querying RegulomeDB
    - Please explain what the argument format is. It is not obvious to non-experts.
    - The last sentence of this sub-section “the output of this function is similar to that used in the queryHaploreg…..” The outputs of queryHaploreg() and queryRegulome() are not similar. The former is a data.base, the latter is a list. Even comparing the data.frame from queryHaploreg() with the first element (res.table) of queryRegulome()  and we found different number of rows, columns, variables and data types (the first contains factors and the second characters). What are the similarities between them?
4. CONCLUSION AND FUTURE WORK: There is not a discussion about the use cases and the conclusions are poor. You should clearly state the advantages to use these packages over the original databases. For example, you could mention the opportunity to generate a more streamlined workflow, shorter retrieval times, a shallow learning curve, etc.
5. SOFTWARE AND DATA AVAILABILITY
   
  - Licence: It is unclear what license the authors use. The authors write GPL-2 | GPL-3, but it is not possible to use both at the same time.
  - Author contributions: The authors mention that IYZ performed evaluation and validation tests. We were expecting these tests to be provided as unit tests. They don’t seem to be included in source code. We suggest to follow best practice by integrating unit tests using the test that framework and using travis-CI (https://travis-ci.org/) for continuous integration. Travis-CI works with Unix base systems, the authors could also test the package on Windows using the appveyor service ( https://www.appveyor.com/).
  - DESCRIPTION file:
    - According to the manual “Writing R extensions”, the description should mention the role of the authors ( https://cran.r-project.org/doc/manuals/r-release/R-exts.html#The-DESCRIPTION-file).
    - The Depends section shows R (>= 3.3). This should be made consistent with the Operation section in which the authors mention to have used R 3.3.2.
  - NAMESPACE file: You seem to use only few functions from the XML and httr packages, so we suggest to load them individually (using importFrom rather than import) to avoid masking.

**Minor comments**

1. ABSTRACT
  - First line of the abstract, “There exists a set of web-based tools for integration and exploring information linked to annotated genetic variants”. We think that this statement would be more appropriate for the introduction because it does not add any key information about the work carried out. The abstract could start with the second sentence, maybe something like, e.g. “This paper presents haploR, a novel R-package ...”
2. INTRODUCTION
  - Second sentence of the fourth paragraph: “The package … downloads results in the form of a data frame or a file”. Technically, a data frame can be saved in a file. Please consider rewording this sentence.
  - The second and the third paragraph could be joined because the topics are strongly related.
3. Grant informations: In most research journals this section is called “Acknowledgments”.

We have read this submission. We believe that we have an appropriate level of expertise to confirm that it is of an acceptable scientific standard, however we have significant reservations, as outlined above.

# Response

**Published:** 2017-05-04

**Authors:** Ilya Zhbannikov

**Affiliations:** Duke University, USA

We thank the reviewers for their careful reading of the manuscript, package testing and their constructive remarks. We have taken the comments on board to improve and clarify the manuscript. Please find below a detailed point-by-point response to all comments (reviewers comments/suggestions (RC) are in italics font; our responses (AR) are in regular, black font.). Unfortunately, due to limited size of the article we could not reflect all the suggestions provided by reviewers explicitly in the article, but we addressed them in corresponding package vignette and web site ( https://github.com/izhbannikov/haploR, README section).

Major comments

INTRODUCTION

**RC1:**

*We think the introduction is rather vague.  There are several sentences such as “in a number of situations” or “in a certain format” which are too vague and require further explanations. For example, instead of saying “in a certain format”,  the authors could explicitly mention the formats that they are referring to (e.g. csv, json, etc).  Again, in the second sentence of the third paragraph “... saving the results of such analyses in different file formats ...”  the authors should again specify what the different file formats are.*

**AR1:**

We rewrote the Introduction section and explicitly mentioned file types. Please also see the package vignette for workflow examples.

**RC2:**

*Just before the fourth paragraph, the authors should mention if this package could be added to one of the CRAN Task Views (https://cran.r-project.org/web/views/) and whether there are other packages with similar goals. If there are other related packages, it would be interesting to mention whether the data could be combined.*

**AR2:**

We added information about other related packages to the Introductory section. haploR is not presented in CRAN Task Views yet but we are working on adding it to there.

METHODS

**RC2:**

*The second sentence of the sub-section Implementation says “Functions….are designed to obtain data from the resources HaploReg...and RegulomeDB….”.  Here, it is important to describe the structure of the retrieved data. We appreciate that most bioinformaticians are familiar with web-based databases such as HaploReg and RegulomeDB.  However, a student might want to use this tool and having a more detailed description of these web databases would be useful to get started. Please, also consider commenting on the use and interpretation of the retrieved information,  for example plotting a subset of the full dataset. The Operation section should include clear instructions for the installation and a complete description  of package dependencies, including versions of the dependent packages.*

**AR2:**

Due to limited space of the article (1,000 words maximum) we provided data description and installation instructions at the package website ( https://github.com/izhbannikov/haploR) and within the corresponding revised vignette ( https://github.com/izhbannikov/haploR/blob/master/vignettes/haplor-vignette.Rmd) or just browseVignettes(“haploR”)).

USE CASES

**RC3**:

*This section is rather vague. The authors should clearly describe all the input arguments of the functions, as well as the expected results. Querying HaploReg - Input vector of SNPs*

**AR3:**

Due to limited size of the paper, we now provide description of the input parameters in the package vignette and the website. Sorry for the inconvenience.

**RC4:**

*When writing example code, it is considered good practice to assign the result of a command to an object, e.g. x <- queryHaploreg(query=c("rs10048158","rs4791078")). Please consider making this change throughout the paper.*

**AR4:**

Thank you for pointing on this. Such issue is fixed in revised article: results of all data retrieval commands are assigned to objects.

**RC5:**

*When we run the command x <- queryHaploreg(query=c("rs10048158","rs4791078")) we get the following message: “No encoding supplied: defaulting to UTF-8”. Consider changing the encoding or removing non-Ascii characters from the table before outputting.*

**AR5:**

We fixed this warning in version 1.4.4 of the package. The parameter encoding added to queryHaploreg function. Default is set to UTF-8.

**RC6:**

*After retrieving the data, please describe the structure of the retrieved object. In particular you should mention the expected number of columns and rows as well as the name and type of variables (the authors might find the str() function useful).*

**AR6:**

We describe this in corresponding vignette due to limited space of the article (not more than 1,000 words). Please see sections **Querying HaploReg**, **Querying RegulomeDB** and their subsections **Output**.

**RC7:**

*We tried to print the object, the result filled the screen and was unreadable.  We suggest to convert the dataframe into a tibble table (see tibble package) to generate a more readable printed output.*

**AR7:**

Thank you for this suggestion. Now we use *tibble* for generating a printable output.

**RC8:**

*We checked the structure of the retrieved objects and the data types are all characters. Some of the columns clearly contain numeric variables (e.g. r2, D , ARF…). We suggest to convert there columns from character to numeric before outputting. This conversion is important because users might incur into errors when generating basic statistics. For instance, running x <- queryHaploreg(query=c("rs10048158","rs4791078"));*

quantile(x$AFR) generates the following error message: “Error in (1 - h) * qs[i] : non-numeric argument to binary operator”.

**AR8:**

This issue is fixed in the current version (1.4.4) of the package available from CRAN. Thank you very much for pointing on that.

**RC9:**

*Querying HaploReg - Input text file with SNPs: This example is reproducible but the authors  do not specify how the "extdata/snps.txt” is structured. We suggest to write something like “the text file should list the rs-IDs in one column, with one rs-ID per row”.*

**AR9:**

We moved this example to the package vignette and package web page where we describe the structure of extdata/snps.txt .

**RC10:**

*Querying HaploReg - Using a particular study: When we extracted the list of studies, we noticed that we cannot subset it using names. Subsetting using indices is prone to errors because the list of studies could increase over time and their order could change.* 

**AR10:**

Thank you for emphasizing this important point. This issue is fixed in 1.4.4 version of the package.

**RC11:**

*Querying RegulomeDB Please explain what the argument format is. It is not obvious to non-experts.*

**AR11:**

We added instructions for the argument format details. Please see package web site README, subsection “Arguments” of section “Querying RegulomeDB” .

**RC12:**

*The last sentence of this sub-section “the output of this function is similar to that used in the queryHaploreg…..” The outputs of queryHaploreg() and queryRegulome() are not similar. The former is a data.base, the latter is a list. Even comparing the data.frame from queryHaploreg() with the first element (res.table) of queryRegulome()  and we found different number of rows, columns, variables and data types (the first contains factors and the second characters). What are the similarities between them?*

**AR12:**

Thank you for this useful remark. We agree that technically these formats are different and similarities are in only the type of information retrieved.

CONCLUSION AND FUTURE WORK:

**RC13:**

*There is not a discussion about the use cases and the conclusions are poor. You should clearly state the advantages to use these packages over the original databases. For example, you could mention the opportunity to generate a more streamlined workflow, shorter retrieval times, a shallow learning curve, etc.*

**AR13:**

We rewrote the conclusion according to your suggestions.

SOFTWARE AND DATA AVAILABILITY

**RC14:**

*Licence: It is unclear what license the authors use. The authors write GPL-2 | GPL-3, but it is not possible to use both at the same time.*

**AR14**:

Thank you for this remark. License changed to GPL-3 in version 1.4.4 of the package.

**RC15:**

*Author contributions: The authors mention that IYZ performed evaluation and validation tests. We were expecting these tests to be provided as unit tests. They don’t seem to be included in source code. We suggest to follow best practice by integrating unit tests using the test that framework and using travis-CI (https://travis-ci.org/) for continuous integration. Travis-CI works with Unix base systems, the authors could also test the package on Windows using the appveyor service (https://www.appveyor.com/).*

**AR15:**

We added unit tests to version 1.4.4 of the package.

DESCRIPTION file:

**RC16:**

*According to the manual “Writing R extensions”, the description should mention the role of the authors (https://cran.r-project.org/doc/manuals/r-release/R-exts.html#The-DESCRIPTION-file).*

**AR16:**

We updated the description file and now it describes the roles of listed contributors.

**RC15:**

*The Depends section shows R (>= 3.3). This should be made consistent with the Operation section in which the authors mention to have used R 3.3.2.*

**AR15:**

We changed the Depends section to R (>= 3.3.2).

**RC16:**

*NAMESPACE file: You seem to use only few functions from the XML and httr packages, so we suggest to load them individually (using importFrom rather than import) to avoid masking.*

**AR16:**

Thank you for this suggestion. Now we import only needed functions with “importFrom” statement.

Minor comments

ABSTRACT

**RC17:**

*First line of the abstract, “There exists a set of web-based tools for integration and exploring information linked to annotated genetic variants”.  We think that this statement would be more appropriate for the introduction because it does not add any key information about the work carried out. The abstract could start with the second sentence, maybe something like, e.g. “This paper presents haploR, a novel R-package ...”*

**AR17:**

Thank you for this helpful suggestion. We adopted the text according to this.

INTRODUCTION

**RC18:**

*Second sentence of the fourth paragraph: “The package … downloads results in the form of a data frame or a file”.  Technically, a data frame can be saved in a file. Please consider rewording this sentence.*

**AR18:**

We reworded this sentence to: "The package connects to the web site, queries the database and downloads results."

**RC19:**

*The second and the third paragraph could be joined because the topics are strongly related.*

**AR19:**

We joined the first and second paragraphs.

**RC20:**

*Grant informations: In most research journals this section is called “Acknowledgments”.*

**AR20:**

We changed the “Grant Information” section name to "Acknowledgments".

# Peer review: Referee response for version 1

**DOI:** 10.5256/f1000research.11583.r19824

**Published:** 2017-02-13

**Authors:** Garrett M. Dancik

**Affiliations:** 1 Department of Computer Science, Eastern Connecticut State University, Willimantic, CT, USA

The authors describe an *R* package named *haploR *for querying the HaploReg and ReglomeDB web-based databases. Because querying can be carried out in *R*, *haploR* adds convenience for querying these databases when subsequent downstream analyses in *R* are desired. 

 The *R* package is easy to use and works as described. However, the potential application of *haploR* is only vaguely described. The authors should include concrete examples of downstream analyses in order to demonstrate when *haploR* would be preferred to traditional queries executed from the web. 

 In addition, addressing the following items would add clarity to the manuscript and the tool:

1. The authors should describe when the results returned by *haploR* differ from the web-based results. For example, whereas the results table from querying HaploReg on the web may indicate that a particular variant has "4 altered motifs", providing links to the variant entry where the motifs are listed, *haploR* directly returns the motifs present. This is an advantage of *haploR* that should be described.
2. There are several spelling and grammatical errors which make the manuscript difficult to follow in some parts. For example, the Introduction states that "Large projects...are devoted to bring together", instead of "bringing together".

I have read this submission. I believe that I have an appropriate level of expertise to confirm that it is of an acceptable scientific standard, however I have significant reservations, as outlined above.

# Response

**Published:** 2017-05-04

**Authors:** Ilya Zhbannikov

**Affiliations:** Duke University, USA

We thank the reviewer for insightful and thorough feedback. It was clear from those comments that our original paper did not emphasize clearly enough the unique contribution of the R package *haploR*. These comments critique helped us to revise the note and package vignette to clarify several aspects of data retrieval methodology used in the package. We revised the paper and this revision addresses all of the reviewer’s concerns. Reviewer comments/suggestions (RC) are in italics font; author’s responses (AR) are in regular, black font.

**RC1:**

*The R package is easy to use and works as described. However, the potential application of haploR is only vaguely described. The authors should include concrete examples of downstream analyses in order to demonstrate when haploR would be preferred to traditional queries executed from the web.*

**AR1:**

We provided corresponding examples in the package vignette and also on the package web page: https://github.com/izhbannikov/haploR . Please see “Motivation and typical analysis workflow” section.

**RC2:**

*In addition, addressing the following items would add clarity to the manuscript and the tool:*

*The authors should describe when the results returned by haploR differ from the web-based results. For example, whereas the results table from querying HaploReg on the web may indicate that a particular variant has "4 altered motifs", providing links to the variant entry where the motifs are listed, haploR directly returns the motifs present. This is an advantage of haploR that should be described.*

**AR2:**

Thank you for this useful suggestion. Following your suggestion and due to limited article size (no more than 1,000 words) we emphasized it in a package vignette (please see the end of “One or several genetic variants” subsection).

**RC3:**

*There are several spelling and grammatical errors which make the manuscript difficult to follow in some parts. For example, the Introduction states that "Large projects...are devoted to bring together", instead of "bringing together".*

**AR3:**

We addressed these errors in the revised article.

We are happy to make any other changes that may be required.

Sincerely,

Ilya Zhbannikov
